# Nanopore Sequencing of a Forensic STR Multiplex Reveals Loci Suitable for Single-Contributor STR Profiling

**DOI:** 10.3390/genes11040381

**Published:** 2020-04-01

**Authors:** Olivier Tytgat, Yannick Gansemans, Jana Weymaere, Kaat Rubben, Dieter Deforce, Filip Van Nieuwerburgh

**Affiliations:** 1 Laboratory of Pharmaceutical Biotechnology, Ghent University, 9000 Gent, Belgium; Olivier.Tytgat@UGent.be (O.T.); Yannick.Gansemans@UGent.be (Y.G.); Jana.Weymaere@UGent.be (J.W.); Kaat.Rubben@UGent.be (K.R.); Dieter.Deforce@UGent.be (D.D.); 2 Department of Life Science Technologies, Imec, 3001 Leuven, Belgium

**Keywords:** forensic, DNA fingerprinting, short tandem repeats, Oxford Nanopore sequencing, massively parallel sequencing

## Abstract

Nanopore sequencing for forensic short tandem repeats (STR) genotyping comes with the advantages associated with massively parallel sequencing (MPS) without the need for a high up-front device cost, but genotyping is inaccurate, partially due to the occurrence of homopolymers in STR loci. The goal of this study was to apply the latest progress in nanopore sequencing by Oxford Nanopore Technologies in the field of STR genotyping. The experiments were performed using the state of the art R9.4 flow cell and the most recent R10 flow cell, which was specifically designed to improve consensus accuracy of homopolymers. Two single-contributor samples and one mixture sample were genotyped using Illumina sequencing, Nanopore R9.4 sequencing, and Nanopore R10 sequencing. The accuracy of genotyping was comparable for both types of flow cells, although the R10 flow cell provided improved data quality for loci characterized by the presence of homopolymers. We identify locus-dependent characteristics hindering accurate STR genotyping, providing insights for the design of a panel of STR loci suited for nanopore sequencing. Repeat number, the number of different reference alleles for the locus, repeat pattern complexity, flanking region complexity, and the presence of homopolymers are identified as unfavorable locus characteristics. For single-contributor samples and for a limited set of the commonly used STR loci, nanopore sequencing could be applied. However, the technology is not mature enough yet for implementation in routine forensic workflows.

## 1. Introduction

Forensic DNA genotyping for identification purposes is routinely performed by analysis of short tandem repeats (STRs) [1]. Those regions in the genome, characterized by short repeating sequences, are polymorphous among individuals regarding the number of repeats. The most commonly applied technique for forensic STR profiling remains polymerase chain reaction (PCR) amplification of STR loci, followed by capillary electrophoresis (CE) fragment size analysis. The importance of massively parallel sequencing (MPS) of STR amplicons increased in recent years [2,3]. Although MPS technologies yield a considerably more informative output, as besides the number of repeats, other types of variation (e.g., single nucleotide polymorphisms (SNPs) and isoalleles) can be assessed. This increased discriminative power is, in particular, beneficial for low-input samples, where dropouts are more common. Moreover, MPS has proven to be of great value for cases where DNA is highly degraded. As only a limited amount of fluorescent dyes can be used for CE analysis, multiple STR markers are labelled with the same dye. The length of the resulting amplicons must not overlap. This restriction is not applicable for MPS technology. Therefore, primer positions can be selected more strategically, resulting in shorter amplicons [4]. Also, more loci can be multiplexed in a single analysis [5,6]. Nevertheless, the high cost involved with MPS due to both the up-front capital investment and the reagent cost, as well as the labor-intensive sample preparation, hamper the widespread adoption of these techniques for forensic identification [7]. 

A drawback of both CE and MPS is the need for bulky equipment, thereby impeding their portability. Shifting from a laboratory-centralized workflow to on-site forensic DNA analysis would connote a range of benefits such as faster results, lower risk for contamination, and less need for trained staff [8]. Oxford Nanopore Technologies (ONT) developed a portable, pocket-sized, USB-powered long-read sequencer, the MinION, which is suited for on-site analysis [9]. Moreover, the up-front cost of the device is negligible compared to other sequencers or a CE-device. The heart of this technology is a disposable flow cell comprising membrane wells, each containing a protein pore. A constant voltage is set across an electrically resistant membrane, giving rise to an ionic current through the orifice. Sequencing is realized by measuring the disruption of this current as single-stranded DNA is forced through the pores. Base calling can be performed using the electrogram, as the alteration of current carries information on the nucleotides present in the pore [10,11]. In contrast to sequencing-by-synthesis methods (e.g., Illumina sequencing), this technology is often classified as third-generation sequencing owing to its applicability for long-read sequencing. Nevertheless, short amplicons can be sequenced with ONT’s technology [12].

Interest in implementing nanopore sequencing is rapidly growing in the forensic field [13]. Evidentially, this should not be at the expense of the quality of DNA profiles. Nanopore sequencing is still considered as a rather new and rapidly evolving technology, thereby continuously improving the data quality. Now and then, a new version of the flow cell is released, with updated pores. The performance of the software used for base calling and further downstream analyses is also crucial, and is the subject of continuous improvements. The platform already evolved in such a way that the MinION sequencer is now capable of correctly genotyping forensic SNP loci [14,15]. The first attempts to assess nanopore sequencing for STR-profiling have been reported recently [16,17]. Unfortunately, results were suboptimal, which can be attributed to multiple factors, both on the chemistry, hardware, and the bioinformatics side. The low signal-to-noise, inhomogeneous translocation speed, and the fact that multiple nucleotides are present simultaneously in the pore are at the base of sequencing errors. An extra complicating factor arises when examining STRs, as these loci are often characterized by the presence of homopolymers. These regions are known to be challenging for base callers as the electrogram signal is constant within homopolymers [18]. 

The goal of this study is twofold. On the one hand, we want to assess the progress made by ONT in the context of sequencing forensic STRs by demonstrating the state of the art. For this purpose, the two most recent versions of flow cells (R9.4 and R10) are compared by analyzing two single-contributor and one mixture sample. R10 is characterized by pores with a longer barrel and a dual reader head, enhancing the accuracy of homopolymer regions. The gathered results are also compared to historical data obtained by our group using the R7 version of MinION flow cells [17]. On the other hand, we want to determine which STR loci are better suited for nanopore sequencing, by identifying characteristics of the STR loci that hinder accurate nanopore sequencing-based STR profiling. These insights might allow the design of a forensic STR panel suitable for nanopore sequencing. Therefore, we included loci with a varying degree of repeat complexity, repeat number, repeat range in the population, and homopolymer content. For this reason, we also include less commonly used STRs like the very complex SE33 locus. The latter features an extremely high repeat number, homopolymers, and a complex structure, and is therefore, not expected to result in an accurate nanopore sequencing-based STR profile.

## 2. Materials and Methods

### 2.1. PCR Amplification

The results presented in this paper were obtained by analyzing three samples, consisting of two single-contributor samples and a mixture sample. Sample A contained reference sample 9947a (OriGene, Rockville, MD, USA), sample B contained reference sample 9948 (OriGene, Rockville, MD, USA), and sample C was a 50:50 mixture sample of those two reference samples. The correct alleles of both reference samples are shown in Table S1. Each sample was subjected to a 15-plex polymerase chain reaction (14 STR-loci and the amelogenin locus for gender identification). All primers were ordered from IDT (Newark, NJ, USA). The primer sequences and concentrations are shown in Table S2. PCR was performed in a total volume of 50 μL, containing 1 ng of input DNA, 1× Qiagen PCR buffer, 0.5 mM MgCl_2_, 0.8 mM dNTPs, and 1.3 U HotStarTaq polymerase (Qiagen, Hilden, Germany). After an initial denaturation step of 15 min at 95 °C, 30 amplification cycles were performed consisting of denaturation for 60 s at 95 °C, primer annealing for 60 s at 59 °C, and elongation for 80 s at 72 °C. A final elongation step of 15 min at 72 °C was performed after thermal cycling. All samples were quantified using the Qubit dsDNA High Sensitivity Assay Kit (Thermo Fisher, Waltham, MA, USA) and size separated by gel electrophoresis (E-gel 2%, Thermo Fisher, Waltham, MA, USA). Amplicons were extracted from the gel (100–400 bp) using the Zymoclean Gel DNA Recovery Kit (Zymo Research, Irvine, CA, USA) in order to remove PCR artifacts, primers, and enzyme. Post-PCR cleanup is not absolutely necessary, but it improves data quality considerably by reducing off-target reads. Of course, other purification methods could also be used, such as silica-based column purification or AMPure XP bead purification. Aliquots of the cleaned PCR products were used for either Illumina sequencing or nanopore sequencing after fluorimetric quantification (Qubit, Thermo Fisher, Waltham, MA, USA).

### 2.2. Illumina Sequencing and Data Analysis

In order to confirm the absence of allelic imbalance, drop-ins, and dropouts originating from the PCR-reaction, the samples were sequenced by Illumina sequencing. This technique is generally considered as the golden standard regarding amplicon MPS and is thereby a valid method for quality control of the PCR. For each sample, 1 μg PCR product was subjected to Illumina sequencing sample preparation using the NEBNext® Ultra™ II DNA Library Prep Kit for Illumina® (NEB, Ipswich, MA, USA) and TruSeq Unique Dual Indexes (UDI) adaptors. Following adaptor ligation, samples were cleaned using the Zymo DNA Clean and Concentrator (Zymo Research, Irvine, CA, USA), according to the manufacturer’s instructions. After library quantification using a Sequencing Library qPCR Quantification kit (Illumina, San Diego, CA, USA) and equimolar pooling of the samples, single-end 300 bp sequencing was performed using a MiSeq Nano Flow Cell (Illumina, San Diego, CA, USA). After demultiplexing of the samples, reads were categorized per locus based on perfect alignment of the primer pairs used to amplify the loci. The categorized reads were aligned against their corresponding reference database of alleles. The alleles encompassed in this database were selected based on their frequency of occurrence within the European population. They are shown in Table S3 [19].

### 2.3. Nanopore Library Preparation and Sequencing

For all samples, two aliquots containing 40 ng of amplicons were subjected to library preparation for Nanopore sequencing. This input corresponds to about 200 fmol, as recommended by ONT. DNA repair and end preparation were performed using NEBNext FFPE DNA Repair Mix and NEBNext End Repair/dA-Tailing Module (NEB, Ipswich, MA, USA). The A-tailed amplicons were cleaned using a 1.8× volume of AMPure XP beads (Beckman Coulter, High Wycombe, UK), followed by quantification using a Qubit fluorimeter. After that, 20 ng of end-prepped amplicons diluted in 22.5 μL nuclease-free water was subjected to a native barcode ligation step, using the Native Barcoding Expansion 1–12 (EXP-NBD104) kit (ONT, Oxford, UK). For this purpose, three different barcodes were used. Ligation of the barcodes was accomplished by adding 25 μL NEB Blunt/TA Ligase Master Mix and 2.5 μL Native Barcode to the sample. After an incubation step of 10 min at room temperature, the barcoded samples were cleaned using a volume of 1.8× AMPure XP beads. The eluate (26 μL) was quantified using a Qubit fluorimeter. From the resulting six barcoded samples, two equimolar pools containing 40 ng of DNA were made. Each pool comprised three barcoded samples, one from each initial PCR mixture. Subsequently, both pools were subjected to an adaptor ligation step, using the SQK-LSK109 kit (ONT, Oxford, UK). For this, 5 μL of Adaptor mix II, 20 μL of NEBNext Quick Ligation Reaction Buffer, and 10 μL of Quick T4 DNA Ligase were added to the pools, followed by an incubation step of 10 min. After adaptor ligation, the library was cleaned using a 1.8× volume of AMPure XP beads. Washing was performed twice using 250 μL Short Fragment Buffer. Beads were resuspended in 15 μL Elution Buffer during a 10 min incubation at room temperature. The eluate (15 μL) was quantified using a Qubit fluorimeter. Before loading on the flow cell, 37.5 μL of Sequencing Buffer and 25.5 μL of Loading Beads were added to 12 μL of the DNA libraries. The first pool was sequenced using a R9.4 flow cell by loading 9 ng (50 fmol) onto the SpotON flow cell, according to the manufacturer’s instruction. The other pool was sequenced using the R10 flow cell. As this flow cell needs a higher input (100 fmol), 18 ng of the second pool was loaded onto the flow cell. To obtain the maximum number of reads possible, both flow cells ran for 48 h. However, most of the reads are obtained during the first hours of the run. The flow cells were controlled and monitored using the MinKNOW software. Every 12 h, the flow cell was refueled with Flush Buffer according to the manufacturer’s instructions. This is best practice for short read sequencing, as this consumes more ATP. ONT is working on a new version of sequencing buffer, capable of regenerating ATP, which should alleviate this intervention [12]. 

### 2.4. Nanopore Data Analysis and Allele Calling

Base calling was performed in real-time during sequencing by the MinKNOW control software (v19.10.1). However, the raw data were re-base called after sequencing using Guppy (v3.4.3) with the polyT scaling option enabled for short reads. Sample demultiplexing based on the barcode sequence was performed by Guppy. For each sample, the reads were subsequently allocated to the correct locus based on the primer sequence using fuzzy regex, allowing two mismatches per primer. The resulting reads were aligned against the same reference allele database as used for the Illumina data, using the Burrows-Wheeler Aligner (BWA, v0.7.17) with the -x ont2d option enabled [20]. The nonaligned reads and the aligned reads having an XA-tag, indicating they aligned to multiple alleles with an equivalent alignment score, were discarded. The number of aligned reads per allele was counted and expressed as the fraction of the total read count of the corresponding STR locus. 

Nanopore sequencing data are noisier as compared to Illumina sequencing data. Therefore, the following rule for allele calling was applied: the allele with the highest number of aligned reads is included, as well as the second most abundant allele, provided that the latter exceeds 50% of the maximum number of aligned reads. This rule cannot be applied for mixture samples, as in forensic casework mixture samples, the contribution of the different contributors is unknown. It was therefore checked whether the true alleles are those with highest fractions of aligned reads.

## 3. Results

### 3.1. Locus Representation Bias

**Figure 1:**
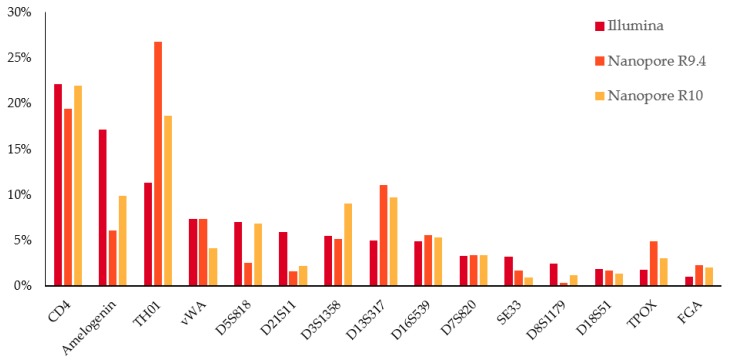
Average distribution of the reads over the loci. Red refers to Illumina data, orange to Nanopore R9.4, and yellow to Nanopore R10.

Illumina sequencing resulted in 316,450 reads, of which 36.6% could be categorized by locus based on the primer sequence. No mismatch in the primer region was allowed for the Illumina data. To assess the amplification bias, the locus representation was calculated as a percentage of the total categorized reads. The results, averaged over the three samples (Figure 1) ordered according to locus representation. The most abundant locus was the CD4 locus, with, on average, 22.05% of the reads. Only 1% of the reads originated from the FGA locus, thereby being the least represented locus. The three most abundant loci (Amelogenin, CD4, and TH01) are among the shortest loci within the multiplex, whereas the least represented loci (FGA, TPOX, and D18S51) belong to the longest loci. 

Respectively, 726,328 and 2,138,545 reads were obtained using the R9.4 and the R10 flow cell. Of these reads, 19.00% and 18.86% of these reads could be categorized by locus based on the primer sequence using fuzzy regex, allowing up to two mismatches per primer. The distribution of categorized nanopore reads over the analyzed loci is also shown in Figure 1. No information was gathered on the average number of mismatches for each primer, as this is only influenced by the primer sequence itself, e.g., the presence/absence of homopolymers.

### 3.2. Alignment of Reads

On average, 99.9% of the locus-categorized Illumina reads aligned uniquely to an allele of the reference database. Table S4 shows the allele representation of Illumina reads. The percentage of reads that aligned to a true allele was 93.68% on average, ranging from 100% (amelogenin) to 65% (SE33) (Table 1). Most of the non-true alignment is due to stutters. Table 1 also shows some key characteristics of the investigated STR loci, such as, the length of homopolymers in the repeat region, the complexity of the repeat pattern (indicating, e.g., compound repeat patterns and noncomplete repeats), and the range of alleles occurring within the population [19].

**Figure 2:**
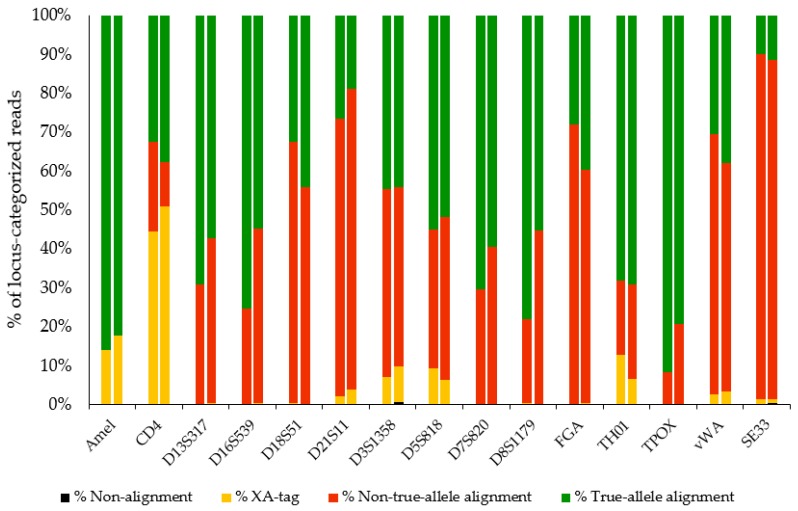
Distribution of locus-categorized reads per locus. Left bars are R9.4 reads and right bars R10. Black refers to non-alignment, yellow to alignment to multiple alleles (XA-tag), red to non-true alignment, and green to true-allele alignment.

On average, 86% (R9.4) and 84% (R10) of the locus-categorized nanopore reads aligned uniquely to an allele of the reference database. The percentage of reads that aligned to a true allele was 56.58% (R9.4) and 54.09% (R10) on average, ranging from 85.97% (amelogenin) to 9.96% (SE33) (Table 1). The remainder of nanopore reads did not align to any allele or aligned to multiple alleles of the reference database (XA-tagged reads). Figure 2 shows this distribution of all locus-categorized nanopore reads, per locus, for both flow cells. 

Among the best-performing STR loci are TPOX, D16S539, D8S1179, D13S317, and TH01, characterized by rather low repeat numbers. However, the locus with the lowest repeat number, CD4, does not perform well. This locus features homopolymeric repeat units (AAAAG). A substantial part of the reads categorized to this locus did not align uniquely. Data quality was increased for this locus using the R10 flow cell. For the SE33 locus, only 10% and 11% of the aligned reads corresponded to one of the true alleles. Other loci that resulted in low-quality reads are vWA, FGA, D21S11, and D18S51, all characterized by a complex repeat pattern.

### 3.3. Nanopore Allele Representation

**Figure 3:**
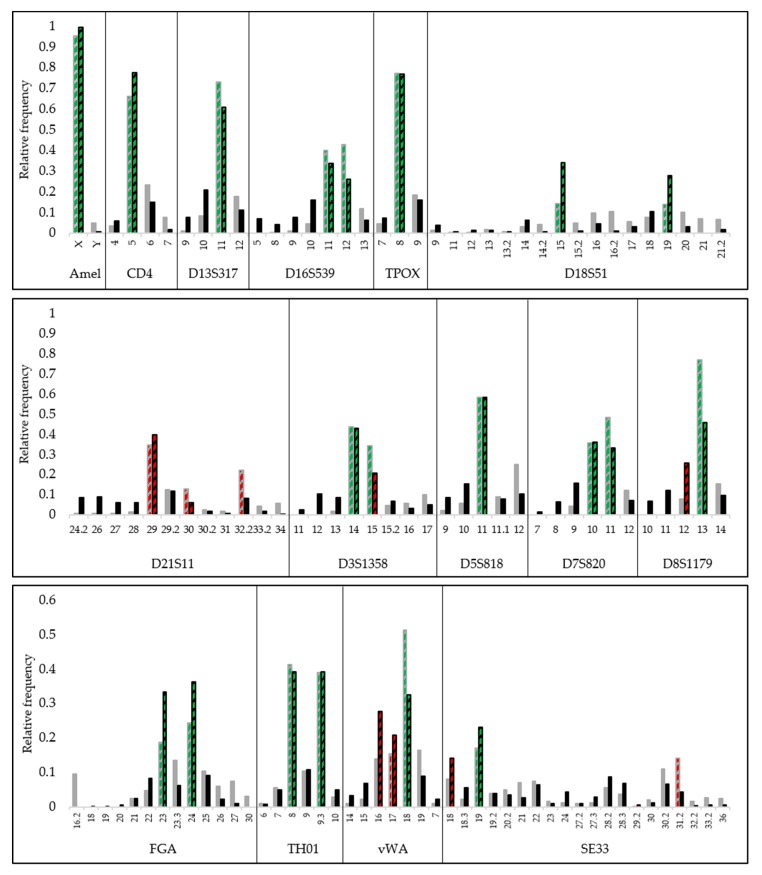
Relative frequency of uniquely aligned reads per locus. Grey indicates R9.4 data and black indicates R10 data. Green-colored bars indicate true positive alleles and red-colored bars indicate drop-ins or dropouts.

The relative frequency of the uniquely aligned reads per locus, obtained using the R9.4 and R10 flow cell is shown for sample A in Figure 3. The results for sample B and sample C are shown in Figures S1 and S2, respectively. From these figures, it can be deduced that the non-true-allele alignment is not only due to stutters but also due to sequencing and alignment errors.

### 3.4. Genotyping

**Figure 4:**
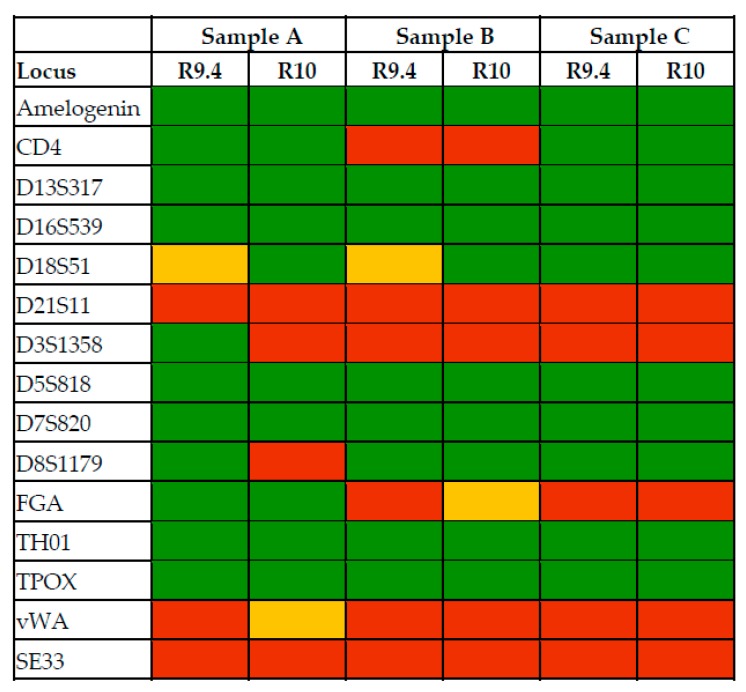
Genotyping results after alignment. Green indicates correct genotyping, red indicates incorrect genotyping, and yellow indicates correct genotyping but complicated by one or more other highly represented alleles.

A summary of the nanopore sequencing-based genotyping results for all samples is shown in Figure 4. Considering all samples and both versions of flow cells, the same loci lead to inaccurate STR genotyping. Between 9 and 11 out of 14 STR loci, as well as the amelogenin locus, were genotyped correctly for all samples. Whether a locus is homozygous or heterozygous for a certain sample did not affect genotyping accuracy.

## 4. Discussion

### 4.1. Amplification and Illumina Sequencing

Amplification was performed using the HotStarTaq enzyme, as this is a commonly used enzyme in the field of forensic genotyping, owing to its high processivity, sensitivity, and robustness. High-fidelity enzymes are more frequently used in combination with DNA sequencing, for obvious reasons. However, the error rate of the detection method used in this study is of another order of magnitude as compared to the error rate of the HotStarTaq enzyme. According to the real-life forensic workflow, a relatively high cycle number was applied during amplification (30 cycles). This could potentially result in more pronounced stutter peaks, amplification bias, and allelic imbalance. To ensure that possible noisy data are not a result of the amplification, an aliquot of amplified DNA was analyzed by Illumina sequencing, resulting in complete profiles with nonexceptional stutter peaks. The observed amplification bias is not unexpected for a 15-plex amplification.

### 4.2. Base Calling and Data-Analysis

Base calling was performed real time during sequencing by the MinKNOW control software (v. 19.10.1). However, the raw data were re-base-called after sequencing using Guppy 3.4.3. This base caller is compatible with the recent R10 flow cell and has a polyT scaling option for short reads. This option ensures scaling of the reads based on the adaptor sequence, whereas for the long reads, scaling is performed based on the read in its entirety [12]. The polyT scaling option was enabled, as scaling based on the complete read might introduce a bias for amplicons with an aberrant GC content.

The aligner used for data analysis is the BWA-MEM aligner, recommended for sequences longer than 70 bp and suited to handle a variety of errors typically observed in third-generation sequencing reads generated by PacBio and nanopore platforms [20]. The recommended -x ont2d option was enabled for all alignments of nanopore reads. Reads that aligned uniquely to a reference allele were filtered by discarding those with an XA tag, indicating alternative mapping locations. This resulted in a moderate loss of data, i.e., 14% of the R9.4 and 20% of the R10 locus-categorized reads. 

### 4.3. Locus-Dependent Characteristics Hampering Accurate Sequencing and Alignment

By assessing the data quality measures for every locus, some locus-dependent characteristics, such as the presence of homopolymers, complex repeat patterns, similarity between the repeating units and the flanking region, and the range of possible alleles, hindering accurate nanopore sequencing and alignment were identified.

Homopolymers are a known cause nanopore sequencing errors, as the presence of a homopolymer in the pore gives rise to a continuous electrogram signal. Efforts are made in order to improve the consensus accuracy for homopolymeric regions, both on the hardware and the software side. An important step towards this goal is the introduction of the R10 flow cell. This version of pores outperformed the R9.4 flow cell for most of the loci characterized by the presence of a homopolymeric region (CD4, D18S51, FGA, and SE33). Another option to tackle the issue of homopolymer sequencing is run-length encoding [21]. In this data-analysis strategy, all strings of identical nucleotides are reduced to one nucleotide, thereby omitting homopolymer indels. However, this is not a valid strategy for some STR loci, due to the occurrence of incomplete repeats in the reference library, e.g., for locus TH01 for which allele 9.3 and allele 10 would become identical.

Complex repeat patterns, such as incomplete repeats or compound repeat patterns, are a major success-limiting characteristic. The worst-performing loci in this study are D21S11, FGA, and SE33, all characterized by complex repeat patterns. These findings are valid for both types of flow cells and are in line with the expectations. Nanopore sequencing is known to generate indels, complicating data analysis of STR loci with complex repeat patterns wherein different alleles can differ by only one or two nucleotides. 

Another potential complicating factor is the sequence of the flanking region. This region can have a sequence very similar to the repeating units, e.g., for the D18S51 locus. The sequence of this flanking region is “AAAGAGAGAGGAAAGAAAGAGAAAAAGAAAAGAAATAGTA”, which is very similar to the repeat of this locus (AGAA). This results in a lowered mapping accuracy. A similar example is the D21S11 locus, whose repeating unit is “TCTA.” The region directly next to the repeating region has the following sequence: “TCGTCTATCTATCCAGTCTATCTA.” The presence of homopolymers in the flanking region does not seem to hamper genotyping. The well-performing D16S539 locus is characterized by multiple homopolymeric regions in the flanking region, posing a challenge to base calling. The effect of these base calling errors will affect the alignment score for all alleles in the reference library to the same extent, and therefore, does not hamper genotyping. No correlation was observed between flanking region length and alignment accuracy.

A final characteristic to consider is the range of alleles occurring in the population, and related to this, the number of repeats. STRs featuring a high repeat number are more prone to both amplification and sequencing errors. Examples are D18S51, SE33, FGA, and D21S11, combining most of the abovementioned characteristics unfavorable for nanopore sequencing, especially the SE33 locus. This locus contains a homopolymeric sequence in the STR, has a high repeat number, has a very complex repeat pattern, and has a wide range of possible alleles. Butler et al. reported an allele range from 3 to 49 repeats, corresponding to 178 observed alleles [22]. Only 65% of the Illumina reads for this locus aligned to a true allele, whereas for all other loci, this fraction was above 85%. As nanopore sequencing is more prone to sequencing errors as compared to Illumina sequencing, these loci cannot be considered for genotyping using ONT technology.

The introduction of the R10 flow cell resulted in higher quality data for loci characterized by homopolymers. However, the technology is still not mature enough for incorporation in a forensic workflow. Besides further improvements on the hardware and software side, designing an STR panel ideally suited for nanopore sequencing proves to be a crucial step. Based on the identified locus-dependent success-limiting characteristics, possible loci on top of the ones identified in this study, that can be considered for inclusion in such a panel are, e.g., CSF1PO, D2S441, D4S2408, D6S1043, D9S1122, D17S1301, D20S482, and D22S1045.

### 4.4. Nanopore Sequencing-Based Genotyping

To demonstrate the state of the art of nanopore sequencing-based forensic genotyping, the two most recent flow cell versions (R9.4 and R10) were compared by analyzing two single-contributor and one mixture sample. For both flow cells, a Gaussian distribution of represented alleles is observed around the true allele (Figure 3, Figures S1 and S2). A fraction of the reads aligned incorrectly to an allele that is one or more repeats longer or shorter. This effect can be explained by the high noise in the nanopore data, caused by sequencing, base calling, and alignment errors. In combination with stutter amplicons present in the sample, this gives rise to the pronounced n − 1 and n + 1 stutters that are more pronounced as compared to a typical CE profile. As the R10 technology is still in its infancy, more random sequencing errors occur for these flow cells, e.g., indels in the repeat region, resulting in even more pronounced stutter peaks.

Amelogenin, D13S317, D16S539, D5S818, D7S820, TH01, and TPOX were genotyped accurately across samples and across flow cell versions. Whether a locus is homozygous or heterozygous for a certain sample did not affect genotyping accuracy. These loci are characterized by simple repeat patterns, low repeat numbers, and the absence of long homopolymers, both in the repeat region as in the remainder of the amplicon. D21S11 D3S1358, FGA, vWA, and SE33 failed consistently across samples and flow cell versions. These samples are characterized by compound or complex repeat patterns, homopolymers, large repeat numbers, and a wide variety of possible alleles within the population. Although the R10 flow cell provided improved data for loci characterized by homopolymers, this was not reflected in improved genotyping of these loci. 

Historical data obtained by our group using the R7 flow cell version resulted in lower genotyping accuracy [17], indicating that advancements were made. Moreover, the yield of sequencing runs is more than a 10-fold higher. At the time of the R7 experiment, amplicons had to be concatenated after PCR in order to obtain DNA of a sufficient length. The current state of the art allows amplicons as short as 80 nucleotides, thereby omitting this extra step during the sample preparation. However, the technology is still not mature enough for incorporation in a standard, routine forensic workflow, even for the selected loci. Currently, it only seems feasible to robustly and accurately genotype single-contributor samples for a limited, selected set of commonly used STR loci.

As discussed in Section 4.3, selecting ideally suited loci is an important step towards implementation of nanopore sequencing in a routine forensic workflow. The next step could be the construction of a dedicated STR panel for this purpose, along with the development of a suitable multiplex amplification reaction. An important aspect will be strategic primer design, by avoiding challenging regions for nanopore sequencing in the primer sequence. This will ensure maximal read recovery. Besides the standard PCR, rolling circle amplification (RCA) promises to be a well-suited alternative as this workflow could increase the genotyping accuracy considerably [23]. In addition, ONT is continuously iterating its technology. A promising example is the development of the R10.3 flow cell, an updated version of the R10 flow cell used in this study [24]. Genotyping based on the raw data, squiggles, has proven its value for large tandem repeats and could potentially be developed for forensic STRs [25].

The price of a single flow cell is another potential cause hindering the implementation of ONT sequencing in the field of forensic genotyping. The recent introduction of Flongle, which is an adaptor for MinION or GridION with 126 pores, is an answer to this issue. Nanopore sequencing has become a very affordable technique, all the more because sample multiplexing is possible.

Although the MinION sequencer is a portable device, sample preparation still requires laboratory equipment, thereby impeding the in-field implementation of this technology. VolTRAX, a sample preparation device which automates all laboratory processes, should meet these needs. The introduction of VolTRAX enables sequencing of DNA samples on remote locations, by minimal trained staff.

## 5. Conclusions

In this study, a forensic STR multiplex on two single-contributor samples and a mixture sample was sequenced using two state-of-the-art versions of Oxford Nanopore flow cells (R9.4 and R10). We demonstrate consistently accurate genotyping for a specific subset of loci, while other loci failed. Although the R10 flow cell provided improved data for loci characterized by homopolymers, this was not reflected in improved genotyping of these loci. Currently, it only seems feasible to robustly and accurately genotype single-contributor samples for a limited, selected set of commonly used STR loci.

Characteristics of the STR loci that hinder accurate nanopore STR sequencing and subsequent mapping to a reference allele database were identified. Repeat number, repeat pattern complexity, flanking region sequence, and the presence of homopolymers still hamper successful genotyping. These findings allow the design of an STR panel ideally suited for nanopore sequencing.

## Figures and Tables

**Table 1:** Percentage of locus-categorized reads that aligned to a true allele and possible mapping—accuracy related characteristics of the STR loci.

	% True-Allele Alignment *			Homopolymer Length (bp)	Complexity Repeat Pattern	Allele Range
Allele	Illumina	R9.4	R10			
Amel	100.00	85.97	82.21	N/A	N/A	X-Y
TPOX	97.16	91.65	79.43	2	Low	8:12
THO1	94.75	68.08	69.26	2	Low	6:10
D7S820	88.47	70.31	59.45	0	Low	7:14
D13S317	89.80	69.11	57.25	0	Low	8:15
D8S1179	93.63	78.21	55.31	0	Medium	8:17
D16S539	91.75	75.36	54.86	0	Low	8:14
D5S818	86.12	55.04	51.91	0	Low	8:14
D3S1358	91.66	44.55	44.20	0	Medium	13:19
D18S51	85.32	32.52	44.10	3	Low	10:22
FGA	81.03	27.95	39.67	3	High	18:28
vWA	84.77	30.54	37.92	0	High	14:20
CD4	97.83	32.62	37.74	4	Low	4:11
D21S11	89.94	26.59	18.87	0	High	27:36
SE33	65.35	9.96	11.44	3	High	12:34
**Total**	**93.68**	**56.58**	**54.09**			

%: Percentage; *: Alignment to stutter alleles is not considered as true alignment.

## Supplementary Materials

The following are available online at https://www.mdpi.com/2073-4425/11/4/381/s1, Table S1: true STR-profile of the examined reference samples as confirmed by capillary electrophoresis, Table S2: forward and reverse primer sequences of the STR multiplex used, Table S3: alleles encompassed in reference database for alignment, Table S4: allele representation of Illumina reads, all samples, Figure S1: relative frequency of aligned reads per locus, sample B, and Figure S2: relative frequency of aligned reads per locus, sample C.
