# Supplementary material for: Nanopore Sequencing of a Forensic STR Multiplex Reveals Loci Suitable for Single-Contributor STR Profiling
Source: Genes (Basel). 2020 Apr 1;11(4):381. doi: 10.3390/genes11040381 (PMC7230633; doi:10.3390/genes11040381)
Supplement: Supplementary file 1 [file genes-11-00381-s001.pdf]

SUPPLEMENTARY DATA

	9947A	9948
**Amelogenin**	X : X	X : Y
**D13S317**	11 : 11	11 : 11
**D16S539**	11 : 12	11 : 11
**D18S51**	15 : 19	15 : 18
**D21S11**	30 : 30	29 : 30
**D3S1358**	14 : 15	15 : 17
**D5S818**	11 : 11	11 : 13
**D7S820**	10 : 11	11 : 11
**D8S1179**	13 : 13	12 : 13
**FGA**	23 : 24	24 : 26
**CD4**	5 : 5	4 : 4
**TH01**	8 : 9.3	6 : 9.3
**TPOX**	8 : 8	8 : 9
**vWA**	17 : 18	17 : 17
**SE33**	19 : 29.2	23.2 : 26.2

Supplementary Table 1: True STR-profile of the examined reference samples as confirmed by capillary electrophoresis.

	Forward primer	Concentration (μM)	Reverse Primer	Concentration (μM)
**Amelogenin**	CCCTGGGCTCTGTAAAGAA	0.5	ATCAGAGCTTAAACTGGGAAGCTG	0.5
**D13S317**	ATTACAGAAGTCTGGGATGTGGAGGA	0.5	GGCAGCCCAAAAAGACAGA	0.5
**D16S539**	GGGGGTCTAAGAGCTTGTAAAAAG	0.8	GTTTGTGTGTGCATCTGTAAGCATGTATC	0.8
**D18S51**	TTCTTGAGCCCAGAAGGTTA	0.5	ATTCTACCAGCAACAACACAAATAAAC	0.5
**D21S11**	ATATGTGAGTCAATTCCCCAAG	0.5	TGTATTAGTCAATGTTCTCCAGAGAC	0.5
**D3S1358**	ACTGCAGTCCAATCTGGGT	0.5	ATGAAATCAACAGAGGCTTGC	0.5
**D5S818**	GGTGATTTTCCTCTTTGGTATCC	0.5	AGCCACAGTTTACAACATTTGTATCT	0.5
**D7S820**	ATGTTGGTCAGGCTGACTATG	0.6	GATTCCACATTTATCCTCATTGAC	0.6
**D8S1179**	ATTGCAACTTATATGTATTTTTGTATTTCATG	1	ACCAAATTGTGTTCATGAGTATAGTTTC	1
**FGA**	GGCTGCAGGGCATAACATTA	0.5	ATTCTATGACTTTGCGCTTCAGGA	0.5
**CD4**	TTACGCGTTTGGAGTCGCAAGCTGAACTAGCG	0.15	CCAGGAAGTTGAGGCTGCAGTGAA	0.6
**TH01**	GTGATTCCCATTGGCCTGTTC	0.5	ATTCCTGTGGGCTGAAAAGCTC	0.5
**TPOX**	GCACAGAACAGGCACTTAGG	0.25	CGCTCAAACGTGAGGTTG	0.25
**vWA**	GCCCTAGTGGATGATAAGAATAATCAGTATGTG	0.5	GGACAGATGATAAATACATAGGATGGATGG	0.5
**SE33**	ACATCTCCCCTACCGCTATA	0.5	AATCTGGGCGACAAGAGTGA	1

Supplementary Table 2: Forward and reverse primer sequences and concentrations used in the STR multiplex PCR reaction.

Locus	Allele	Locus	Allele	Locus	Allele	Locus	Allele	Locus	Allele	Locus	Allele	Locus	Allele
Amelogenin	X	D7S820	7	D21S11	26	D16S539	5	D8S1179	10	SE33	7	SE33	26.2
	Y		8		27		6		11		8		27
CD4	4		9		28		7		12		9		27.2
	5		10		29		8		13		10		27.3
	6		11		29.2		9		14		10.2		28.2
	7		12		30		10		15		11		28.3
	8		13		30.2		11		16		12		29.2
	9		14		30.3		12		17		13		30
	10	D18S51	9		31		13		18		13.2		30.2
	11		10		31.2		14	FGA	18		15.2		31
D16S317	7		11		32		15		19		16		31.2
	8		12		32.2		16		20		17		32
	9		13		33	TPOX	6		21		17.2		32.2
	10		13.2		33.1		7		22		17.3		33
	11		14		33.2		8		23		18		34
	12		14.2		34		9		23.3		18.3		34.2
	13		15		35		10		24		19		35
	14		15.2		36		11		25		19.2		36
	15		16		37	vWA	11		26		20		
D5S818	7		16.2		38		12		27		20.2		
	8		17		39		13	D3S1358	11		21		
	9		18	TH01	5		14		12		21.2		
	10		19		6		15		13		22		
	11		20		7		16		14		22.2		
	12		21		8		17		15		23		
	13		21.2		9		18		16		23.2		
	14		22		9.3		19		17		24		
	15		23		10		20		18		24.2		
	16		24		11		21		19		25.2		
									20		26		

Supplementary Table 3: Alleles encompassed in reference database for alignment.

Locus	Allele	Sample A	Sample B	Sample C	Locus	Allele	Sample A	Sample B	Sample C
Amel	X	100%	100%	100%	D8S1179	11	1%	6%	4%
	Y	0%	94%	27%		12	10%	100%	37%
CD4	4	2%	100%	59%		13	100%	80%	100%
	5	100%	0%	100%		14	1%	0%	1%
D13S317	9	1%	1%	1%	FGA	16.2	7%	10%	0%
	10	9%	10%	9%		22	13%	10%	0%
	11	100%	100%	100%		23	100%	10%	75%
	12	1%	1%	1%		24	73%	100%	100%
D16S539	8	0%	1%	2%		25	0%	0%	0%
	9	1%	0%	2%		26	7%	30%	25%
	10	10%	9%	14%		27	0%	0%	25%
	11	100%	100%	100%		28	0%	10%	0%
	12	76%	0%	33%		29	7%	0%	0%
D18S51	13	2%	0%	0%	TH01	5	1%	5%	2%
	14	14%	11%	13%		6	1%	94%	36%
	15	100%	100%	100%		7	5%	1%	4%
	17	0%	11%	8%		8	96%	0%	68%
	18	5%	53%	29%		9	4%	3%	4%
	19	34%	0%	54%		9.3	100%	100%	100%
D21S11	27	0%	2%	1%		10	1%	1%	1%
	28	2%	11%	5%	TPOX	7	3%	4%	5%
	29	16%	100%	41%		8	100%	100%	100%
	30	100%	77%	100%		9	0%	84%	15%
	31	1%	2%	1%	vWA	15	3%	3%	3%
D3S1358	12	1%	0%	1%		16	16%	15%	15%
	13	9%	1%	7%		17	100%	100%	100%
	14	100%	11%	74%		18	73%	1%	39%
	15	88%	100%	100%		19	1%	0%	1%
	16	0%	14%	7%		20	1%	0%	0%
	17	0%	81%	37%	SE33	18	18%	0%	18%
D5S818	7	2%	5%	4%		19	100%	1%	100%
	8	0%	1%	1%		20	5%	1%	7%
	9	2%	2%	1%		21	1%	1%	1%
	10	10%	11%	10%		22	2%	0%	4%
	11	100%	100%	100%		22.2	1%	11%	8%
	12	1%	14%	4%		23	1%	2%	6%
	13	0%	96%	26%		23.2	0%	100%	68%
	14	0%	1%	1%		24	1%	0%	0%
D7S820	8	1%	0%	1%		24.2	1%	4%	4%
	9	8%	2%	4%		25.2	1%	11%	9%
	10	100%	9%	54%		26	1%	0%	0%
	10.3	6%	9%	8%		26.2	0%	57%	48%
	11	86%	100%	100%		27.2	3%	5%	4%
	13	0%	0%	0%		28.2	7%	4%	13%
	14	0%	0%	0%		29.2	55%	1%	29%

Supplementary Table 4: Allele representation by Illumina reads for all samples. Grey indicates true alleles.

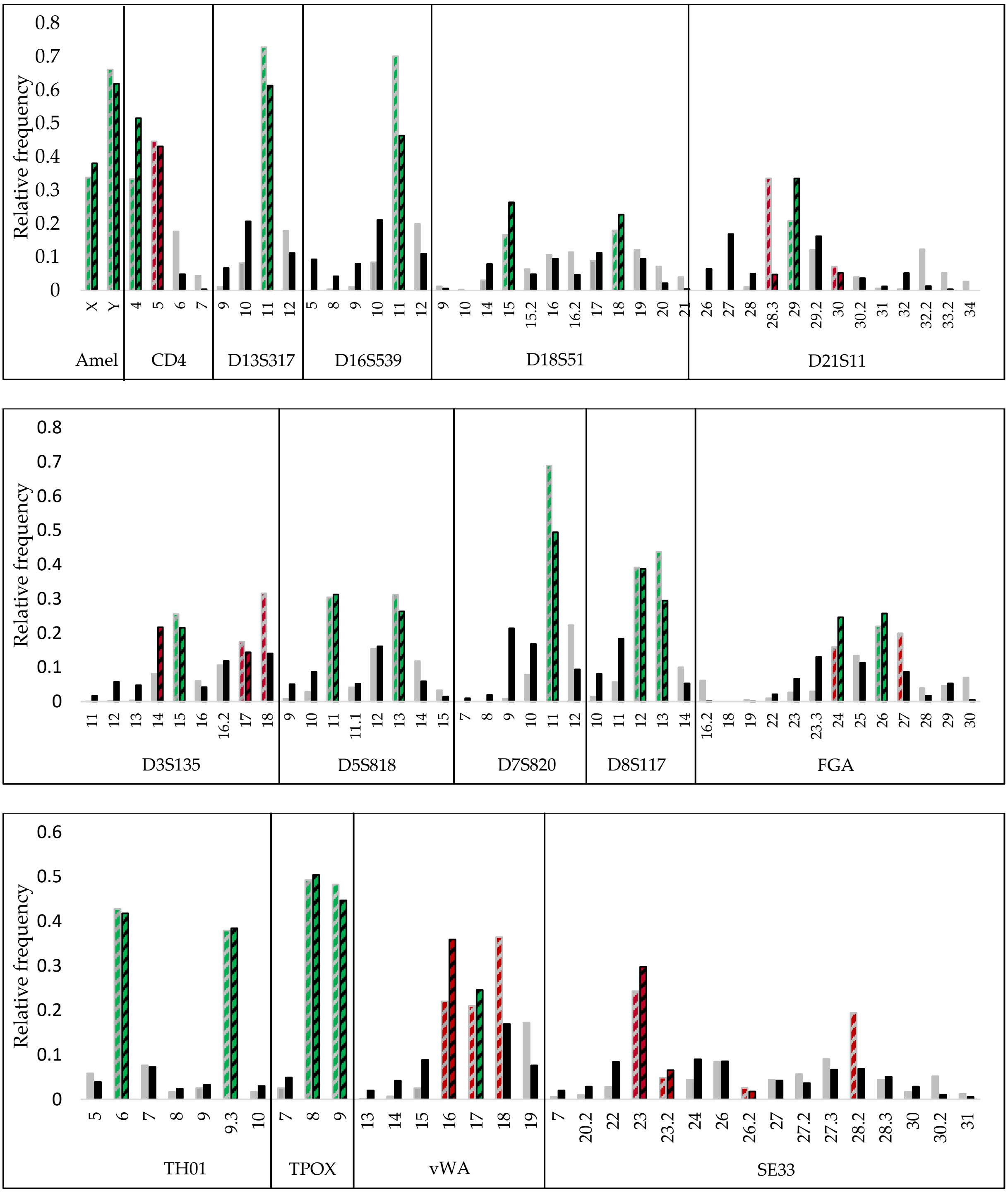

Supplementary Figure 1: Relative frequency of aligned reads per locus, sample B. Grey indicates R9.4 data, black indicates R10 data. Green colored bars indicate true positive alleles, red colored bars indicate drop-ins or drop-outs.

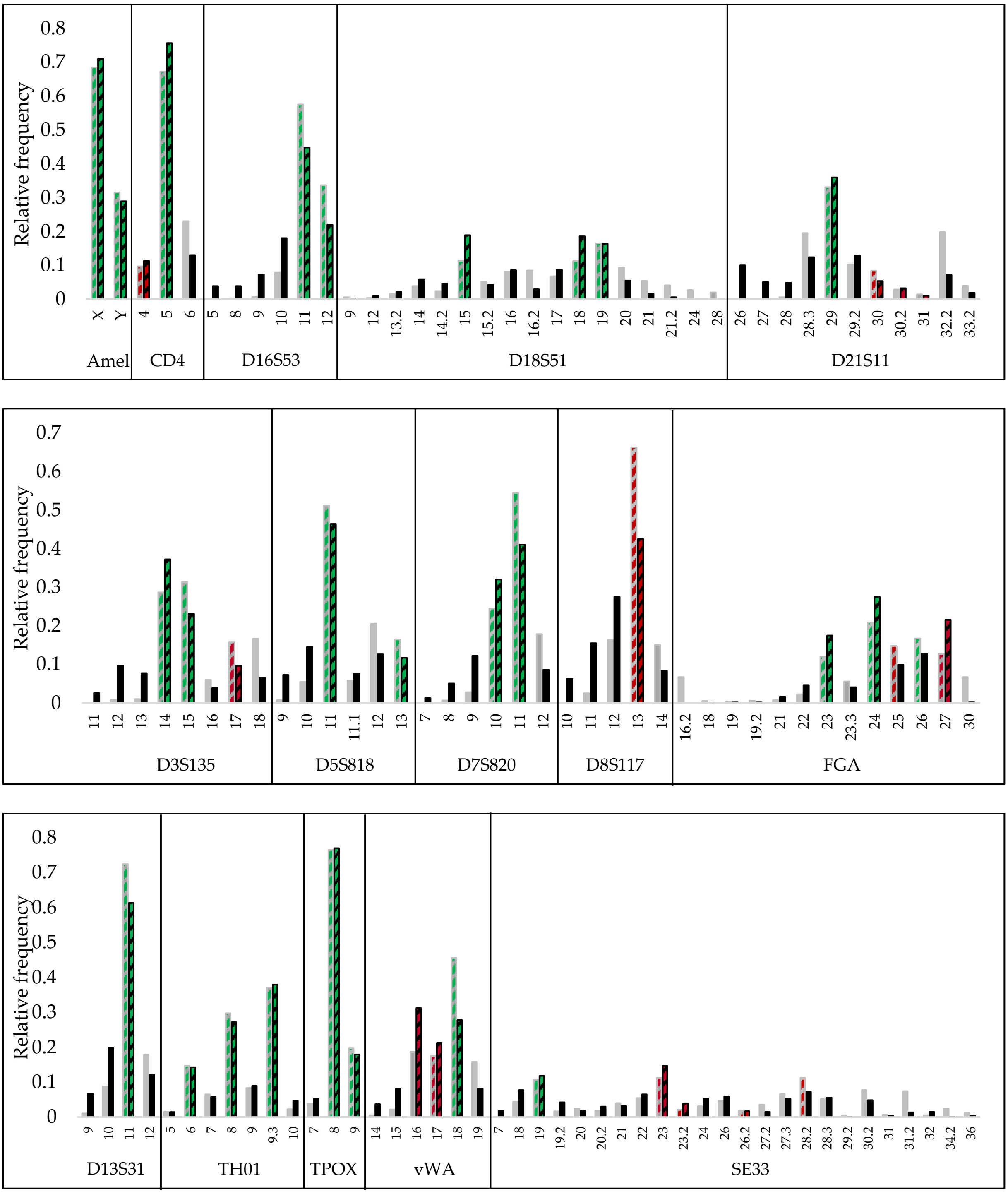

Supplementary Figure 2: Relative frequency of aligned reads per locus, sample C. Grey indicates R9.4 data, black indicates R10 data. Green colored bars indicate true positive alleles, red colored bars indicate drop-ins or drop-outs.
